# Genetic variants modify the effect of age on *APOE* methylation in the Genetics of Lipid Lowering Drugs and Diet Network study

**DOI:** 10.1111/acel.12293

**Published:** 2014-12-04

**Authors:** Yiyi Ma, Caren E Smith, Chao-Qiang Lai, Marguerite R Irvin, Laurence D Parnell, Yu-Chi Lee, Lucia Pham, Stella Aslibekyan, Steven A Claas, Michael Y Tsai, Ingrid B Borecki, Edmond K Kabagambe, Silvia Berciano, José M Ordovás, Devin M Absher, Donna K Arnett

**Affiliations:** 1 Nutrition and Genomics Laboratory, Jean Mayer US Department of Agriculture Human Nutrition Research Center on Aging at Tufts UniversityBoston, MA, USA; 2 Department of Epidemiology, University of Alabama at BirminghamBirmingham, AL, USA; 3 Department of Laboratory Medicine and Pathology, University of MinnesotaMinneapolis, MN, USA; 4 Department of Genetics, Washington University School of MedicineSt. Louis, MO, USA; 5 Department of Medicine, Vanderbilt UniversityNashville, TN, USA; 6 Instituto Madrileño de Estudios Avanzados en Alimentación (IMDEA-FOOD)Madrid, Spain; 7 Department of Epidemiology, Centro Nacional Investigaciones Cardiovasculares (CNIC)Madrid, Spain; 8 Hudson Alpha Institute for BiotechnologyHuntsville, AL, USA

**Keywords:** apolipoprotein E, age, DNA methylation, variants, epidemiology, interaction

## Abstract

Although apolipoprotein E (*APOE*) variants are associated with age-related diseases, the underlying mechanism is unknown and DNA methylation may be a potential one. With methylation data, measured by the Infinium Human Methylation 450 array, from 993 participants (age ranging from 18 to 87 years) in the Genetics of Lipid Lowering Drugs and Diet Network (GOLDN) study, and from Encyclopedia of DNA Elements (ENCODE) consortium, combined with published methylation datasets, we described the methylation pattern of 13 CpG sites within *APOE* locus, their correlations with gene expression across cell types, and their relationships with age, plasma lipids, and sequence variants. Based on methylation levels and the genetic regions, we categorized the 13 *APOE* CpG sites into three groups: Group 1 showed hypermethylation (> 50%) and were located in the promoter region, Group 2 exhibited hypomethylation (< 50%) and were located in the first two exons and introns, and Group 3 showed hypermethylation (> 50%) and were located in the exon 4. *APOE* methylation was negatively correlated with gene expression (minimum *r *=* *−0.66, *P *=* *0.004). *APOE* methylation was significantly associated with age (minimum *P *=* *2.06E-08) and plasma total cholesterol (minimum *P *=* *3.53E-03). Finally, *APOE* methylation patterns differed across *APOE* ε variants (minimum *P *=* *3.51E-05) and the promoter variant rs405509 (minimum *P *=* *0.01), which further showed a significant interaction with age (*P *=* *0.03). These findings suggest that methylation may be a potential mechanistic explanation for *APOE* functions related to aging and call for further molecular mechanistic studies.

## Introduction

Apolipoprotein E (ApoE, encoded by the *APOE* gene), a protein involved in both exogenous and endogenous lipid metabolism, plays a significant role in the process of age-related diseases, including cardiovascular diseases, Alzheimer's disease, and age-related macular disease (Ang *et al*., 2008). While the vast majority of studies have investigated relationships between *APOE* sequence variants and age-related diseases (Davignon *et al*., 1988; Saunders *et al*., 1996; Klaver *et al*., 1998; Ward *et al*., 2009), current studies on the relationship between aging and methylation pattern *APOE* are limited. One small-scale study suggested that the differences in *APOE* methylation between brains with late-onset Alzheimer's disease and normal brains increase with age (Wang *et al*., 2008). The effect of aging on *APOE* methylation is highly plausible based on the general link between DNA methylation and aging. For example, nearly every step of cellular development and differentiation involves DNA methylation changes (Cedar & Bergman, 2012). DNA methylation has also been shown to be associated with age-related diseases (Johnson *et al*., 2012). Furthermore, methylation of *APOE* was shown to be functional as it is modified by environmental factors such as folate (Yi-Deng *et al*., 2007; Glier *et al*., 2014) and correlates with clinical phenotypes in some (Turan *et al*., 2012) but not all studies (Sharma *et al*., 2008).

In light of the potential shared relationship with aging that appears to link *APOE* sequence variants and *APOE* methylation, along with the observation that sequence variants may actually alter methylation status (Zhi *et al*., 2013), we propose that studying the two phenomena in combination may be especially informative. Specifically, three functional single nucleotide polymorphisms (SNPs) may modify DNA methylation at the *APOE* locus. The first SNP, rs405509, is located in the promoter region. The variant of this SNP was postulated to increase DNA methylation based on its demonstrated decreasing effect on gene transcription (Artiga *et al*., 1998), the main functional effect of DNA methylation (Lindahl, 1981). The other two SNPs are rs429358 and rs7412, which define the ε2/ε3/ε4 isoforms of ApoE and both are located within exon 4. These two SNPs are hypothesized to change DNA methylation as well, not only because they are located within the CpG island contained in the exon 4, but also because both are CpG-related SNPs (the cytosine allele forms a CpG dinucleotide while the thymine allele disrupts it).

Using data from 993 participants of the Genetics of Lipid Lowering Drugs and Diet Network (GOLDN) study, publically available data from Encyclopedia of DNA Elements (ENCODE) consortium, and previously open-published datasets, we described the general methylation pattern of the 13 CpG sites distributed along the entire *APOE* locus, which were available in the Infinium Human Methylation 450 array. With the data from ENCODE consortium, we analyzed the relationship between *APOE* methylation and gene expression across different cell types. Finally, we utilized the GOLDN population to explore (i) whether age is associated with *APOE* methylation, (ii) whether the effects of age on *APOE* methylation leads to changes in plasma lipids, the main functional phenotype of *APOE*, and (iii) whether the effect of age on *APOE* methylation can be modulated by methylation-related genetic variants in the *APOE* locus.

## Results

### Population characteristics of GOLDN by age

Population characteristics were compared across age quintiles (Table1). Gender distribution did not differ across age quintiles. Compared to the younger age quintiles, the older quintiles tended to contain fewer smokers (*P *=* *0.004) but more individuals with a past history of taking antilipemic medications (*P *<* *0.0001). Also, those individuals in the older age quintiles tended to consume less total energy, vitamin B12, and folate (*P *≤* *0.0001).

**Table 1:** Subject characteristics of GOLDN by age quintiles*

	Age quintiles					
Variable	Q1	Q2	Q3	Q4	Q5	*P* for trend†
Age (year)‡	24 (18–34)	40 (35–43)	47 (44–51)	57 (52–64)	71 (65–87)	–
*N*	200	185	204	209	195	–
Men	88 (44.0)	98 (53.0)	96 (47.1)	99 (47.4)	94 (48.2)	0.64
Current smoker	17 (8.5)	18 (9.7)	21 (10.3)	13 (6.2)	4 (2.1)	0.004
Ever drinker	79 (39.5)	101 (54.6)	104 (51.0)	102 (48.8)	95 (48.7)	0.16
Total energy intake (kcal day^−1^)	2448 (1758)	2228 (1202)	2169 (981)	2037 (925)	1746 (809)	< 0.0001
Vitamin B12 intake (μg day^−1^)	6.1 (4.4)	5.4 (3.3)	5.3 (3.0)	5.3 (2.9)	4.7 (3.3)	0.0001
Folate intake (μg day^−1^)	467.0 (315.7)	411.9 (237.7)	398.2 (171.4)	398.5 (187.1)	362.9 (166.5)	< 0.0001
History of taking antilipemic medications	0 (0.0)	2 (1.1)	1 (0.5)	14 (6.7)	20 (10.3)	< 0.0001

* Data are means (standard deviation) or *n* (%).

† Mantel–Haenszel χ^2^ tests and ANOVA tests were applied to obtain *P*-values for trend of categorical and continuous variables, respectively, according to the median of age in each quintile.

‡ Data are median age (minimum age–maximum age) within each quintile.

### *APOE* methylation patterns are similar in blood lymphocytes and other cell types

**Figure 1:**
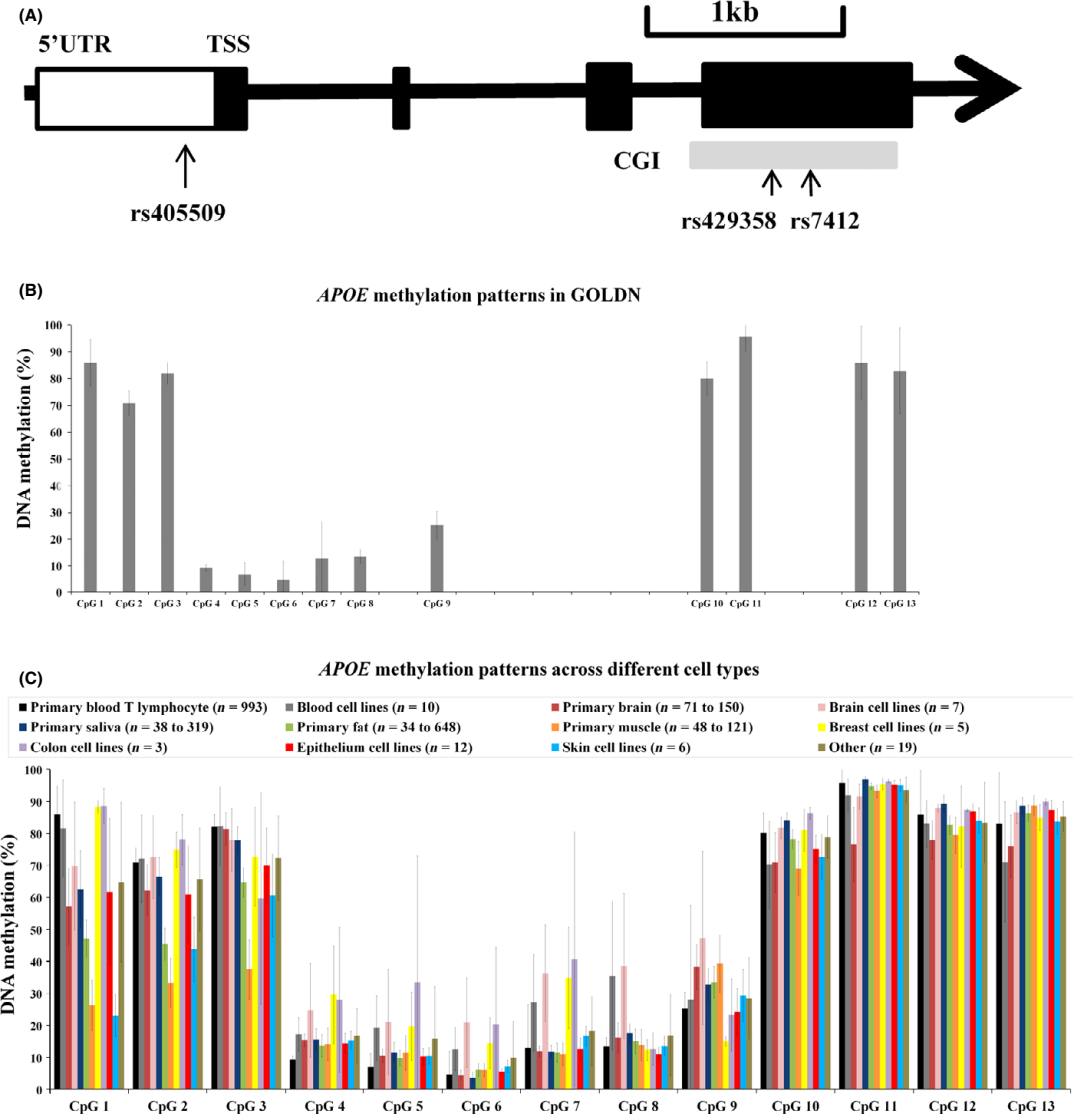
Gene structure and methylation pattern of *APOE* measured in blood T lymphocyte in GOLDN and other cell types in public datasets. Panel A shows the structure of *APOE* gene. The arrow represents the direction of the gene, and filled rectangles represent exons. Panels B and C show the mean of *APOE* methylation (%) for each of the 13 CpG sites measured in blood T lymphocytes in GOLDN and across different cell types in public datasets.

We examined the methylation status of 13 CpG sites distributed along the entire *APOE* using data from GOLDN, ENCODE, and previous publications. The genetic structure of *APOE* locus is illustrated in Fig.1A. Based on both the methylation levels in GOLDN and genetic locations (Fig.1B), three groups of CpG sites can be distinguished. Specifically, the first three CpG sites comprised a group (Group 1) that was both hypermethylated (all sites > 50% methylation) and located within the promoter region. The second group of CpG sites (Group 2, sites 4–9) was both hypomethylated (all sites < 50%) and located in the 5′ part of the gene. The third group (Group 3, sites 10–13) was both hypermethylated (all sites > 50%) and located at the 3′ end of the gene. This categorization of the 13 CpG sites into three groups was also suggested by the heat map of GOLDN (Figure S1).

To explore the generalizability of the blood T lymphocyte used in GOLDN, we compared the *APOE* methylation patterns of these 13 CpG sites found in GOLDN (presented as the first bar in Fig.1C) with those measured across different cell types (presented as the bars starting from the second in Fig.1C), including primary cells from tissues of brain, saliva, fat, muscle, and liver, and 61 cell lines from tissues of brain, blood, breast, colon, epithelia, skin, muscle, heart, kidney, lung, ovary, pancreas, blood vessel, and prostate (Table S1). In general, *APOE* methylation patterns are similar across different cell types by consistently showing that the methylation levels of the CpG sites within Group 2 are lower than those of the sites located within Group 1 and Group 3. Consistently, across all the cell types, CpG sites within Group 3 have hypermethylation levels (all sites > 50%) and those sites in Group 2 have hypomethylation levels (almost all sites < 50%). Methylation levels of the CpG sites within Group 1 showed variation across cell types, which have hypermethylation (> 50%) in tissues of brain and saliva, but hypomethylation (< 50%) in tissues of fat, muscle, and skin. Despite these variations in Group 1, cell type-specific heat maps consistently suggested that these 13 CpG sites can be categorized into three distinct groups (Figures S2–S6).

### *APOE* methylation patterns are correlated with *APOE* gene expression

We extended our analyses in ENCODE by evaluating the relationship between CpG methylation and gene expression across 17 cell types with data available for both gene expression and methylation. Methylation of four CpG sites, distributed across three methylation groups identified above, was negatively correlated with gene expression (Table2). Two CpG sites within Group 1, CpG 2 (cg04406254) and CpG 3 (cg01032398), showed the strongest correlation with gene expression, with Pearson's correlation coefficients of −0.66 (*P *=* *0.004) and −0.62 (*P *=* *0.008), respectively. In Group 2, CpG 7 (cg18768621) had a borderline significance (*P *=* *0.05) with a coefficient of −0.48. In Group 3, CpG 12 (cg18799241) was negatively correlated with a coefficient of −0.51 (*P *=* *0.04).

**Table 2:** Correlations between *APOE* methylation and *APOE* gene expression in ENCODE

CpG group*	CpG #	CpG name	Pearson's correlation coefficients	*P*
Group 1	CpG 1	cg14123992	−0.45	0.07
	CpG 2	cg04406254	−0.66	0.004
	CpG 3	cg01032398	−0.62	0.008
Group 2	CpG 4	cg26190885	−0.17	0.52
	CpG 5	cg12049787	−0.33	0.20
	CpG 6	cg08955609	−0.16	0.53
	CpG 7	cg18768621	−0.48	0.05
	CpG 8	cg19514613	−0.40	0.11
	CpG 9	cg06750524	−0.28	0.28
Group 3	CpG 10	cg16471933	0.39	0.12
	CpG 11	cg05501958	0.21	0.43
	CpG 12	cg18799241	−0.51	0.04
	CpG 13	cg21879725	0.30	0.25

* CpG groups were defined according to both the methylation level and region of the gene.

### *APOE* methylation patterns are associated with age

We next evaluated relationships between age and *APOE* methylation by examining methylation of the 13 CpG sites occurring within the three distinct methylation groups (Group 1, Group 2, and Group 3), as described above. Age was significantly associated with methylation of at least one CpG site in each group (Table3). For example, age was significantly negatively associated with DNA methylation values for all three CpG sites in Group 1 including CpG 1 (cg14123992), CpG 2 (cg04406254), and CpG 3 (cg01032398) (*P = *8.00E-05, 2.06E-08, and 1.16E-04, respectively). However, age was significantly positively associated with methylation of CpG 8 (cg19514613) (*P *=* *0.004), one CpG site in Group 2. For Group 3, age was significantly negatively associated with methylation of CpG 10 (cg16471933) (*P *=* *0.04). Significant relationships were not altered in the secondary analyses adjusted for smoking, drinking, total energy intake, physical activity, vitamin B12 intake, folate intake, hormone replacement therapy in women, history of taking antilipemic medications, and time of fasting blood drawn (data not shown).

**Table 3:** *APOE* methylation patterns and plasma lipids by age quintiles in GOLDN*

				Age quintiles†					
				Q1	Q2	Q3	Q4	Q5	
				24 (18–34) years	40 (35–43) years	47 (44–51) years	57 (52–64) years	71 (65–87) years	
Outcomes	CpG group	CpG #	CpG name	*n *= 200	*n *= 185	*n *= 204	*n *= 209	*n *= 195	*P* ‡
*APOE* methylation (%)	Group 1	CpG 1	cg14123992	88.13 (0.45)	85.32 (0.83)	86.07 (0.61)	85.72 (0.42)	84.46 (0.62)	8.00E-05
		CpG 2	cg04406254	72.36 (0.24)	70.62 (0.35)	70.75 (0.33)	70.29 (0.34)	69.90 (0.27)	2.06E-08
		CpG 3	cg01032398	82.80 (0.18)	81.58 (0.45)	82.22 (0.22)	81.96 (0.23)	81.39 (0.23)	1.16E-04
	Group 2	CpG 4	cg26190885	9.17 (0.10)	9.30 (0.09)	9.32 (0.08)	9.44 (0.08)	9.33 (0.08)	0.11
		CpG 5	cg12049787	6.63 (0.24)	7.13 (0.42)	6.94 (0.35)	6.80 (0.19)	7.02 (0.23)	0.44
		CpG 6	cg08955609	3.79 (0.11)	5.38 (0.77)	4.83 (0.59)	4.69 (0.44)	4.69 (0.42)	0.19
		CpG 7	cg18768621	11.39 (0.86)	14.77 (1.23)	11.98 (0.81)	12.76 (1.12)	13.70 (1.10)	0.30
		CpG 8	cg19514613	13.13 (0.15)	13.21 (0.22)	13.40 (0.15)	13.92 (0.20)	13.61 (0.18)	0.004
		CpG 9	cg06750524	24.78 (0.25)	25.75 (0.51)	25.51 (0.42)	25.35 (0.35)	24.66 (0.27)	0.60
	Group 3	CpG 10	cg16471933	80.97 (0.35)	79.51 (0.49)	80.01 (0.50)	80.28 (0.37)	79.54 (0.39)	0.04
		CpG 11	cg05501958	96.29 (0.11)	95.17 (0.60)	95.41 (0.50)	95.65 (0.37)	95.98 (0.09)	0.62
		CpG 12	cg18799241	87.27 (0.87)	83.57 (1.27)	86.23 (0.86)	86.49 (1.08)	85.62 (1.08)	0.68
		CpG 13	cg21879725	84.22 (1.15)	80.46 (1.37)	83.64 (1.02)	83.74 (1.20)	82.12 (1.24)	0.62
Plasma lipids	TC (mg dL^−1^)			164.10 (2.81)	186.92 (2.37)	191.59 (2.21)	198.47 (2.57)	188.96 (2.87)	1.04E-07
	LDL-c (mg dL^−1^)			104.09 (2.57)	120.23 (2.20)	121.90 (1.91)	127.20 (2.20)	116.65 (2.32)	2.00E-04
	HDL-c (mg dL^−1^)			46.32 (0.90)	48.58 (0.85)	48.63 (0.90)	48.74 (0.94)	49.63 (0.95)	0.01
	TG (mg dL^−1^)			87.02 (1.05)	105.94 (1.05)	114.35 (1.05)	132.17 (1.04)	127.43 (1.05)	1.94E-08

* Data are the least square means (standard error of the means) of outcomes adjusted for the covariates below;

† Median age (minimum age–maximum age) within each quintile were presented;

‡ *P*:*P*-value for the association of age (quintile) with *APOE* methylation patterns and plasma lipids, adjusting for pedigree, sex, center, and the first principal component of cellular purity and population structure.

### *APOE* methylation patterns may act as the intermediate factors of the effects of age on blood lipids

We confirmed the known effects of age on plasma lipids in GOLDN (Table3) by showing that those individuals in the older age quintiles tended to have higher total cholesterol (TC) (*P *=* *1.04E-07), low-density lipoprotein cholesterol (LDL-c) (*P *=* *2.00E-04), high-density lipoprotein cholesterol (HDL-c) (*P *=* *0.01), and triglycerides (TG) (*P *=* *1.94E-08).

We also found that *APOE* methylation patterns are associated with plasma lipids of TC and LDL-C, the main phenotypes of *APOE* (Table4). Methylations of six CpG sites from all three CpG groups were significantly associated with plasma TC. Specifically, methylations of CpG sites in Group 1 and Group 2, which were CpG 1 (cg14123992), CpG 2 (cg04406254), CpG 10 (cg16471933), CpG 12 (cg18799241), and CpG 13 (cg21879725), were negatively associated with TC (*P *<* *0.05). Methylation of the CpG site in Group 2, CpG 7 (cg18768621), was positively associated with TC (*P *=* *0.02). For LDL-c, methylation of CpG 9 (cg06750524) had a significantly positive association (*P *=* *0.01).

**Table 4:** Associations between *APOE* methylation patterns and plasma lipids in GOLDN

			TC			LDL-c			HDL-c			TG (log)		
CpG group	CpG #	CpG name	Coefficient*	SEM*	*P* †	Coefficient*	SEM*	*P* †	Coefficient*	SEM*	*P* †	Coefficient‡	SEM‡	*P* †
Group 1	CpG 1	cg14123992	−0.32	0.10	3.53E−03	−0.20	0.11	0.07	−0.10	0.05	0.08	−0.001	0.002	0.53
	CpG 2	cg04406254	−0.56	0.20	0.02	−0.34	0.18	0.10	−0.08	0.08	0.38	−0.01	0.005	0.09
	CpG 3	cg01032398	−0.12	0.23	0.61	0.05	0.20	0.81	−0.14	0.08	0.24	0.001	0.01	0.87
Group 2	CpG 4	cg26190885	0.37	0.89	0.67	0.25	0.75	0.74	0.29	0.31	0.35	0.01	0.01	0.72
	CpG 5	cg12049787	0.36	0.20	0.06	0.24	0.21	0.25	0.15	0.12	0.24	−0.002	0.004	0.63
	CpG 6	cg08955609	0.14	0.13	0.30	0.16	0.13	0.26	0.03	0.04	0.45	−0.001	0.003	0.68
	CpG 7	cg18768621	0.20	0.08	0.02	0.13	0.07	0.07	−0.01	0.03	0.63	0.003	0.001	0.06
	CpG 8	cg19514613	−0.30	0.43	0.50	0.07	0.38	0.86	−0.12	0.16	0.44	−0.002	0.01	0.77
	CpG 9	cg06750524	0.35	0.17	0.06	0.45	0.15	0.01	−0.02	0.06	0.70	0.001	0.00	0.74
Group 3	CpG 10	cg16471933	−0.49	0.15	0.01	−0.24	0.14	0.11	−0.01	0.07	0.93	−0.01	0.004	0.08
	CpG 11	cg05501958	−0.28	0.12	0.09	−0.29	0.11	0.09	−0.04	0.05	0.39	−7.70E−05	0.003	0.98
	CpG 12	cg18799241	−0.19	0.07	0.02	−0.11	0.06	0.12	−0.01	0.03	0.76	−0.002	0.002	0.19
	CpG 13	cg21879725	−0.19	0.06	0.008	−0.11	0.06	0.06	0.004	0.02	0.86	−0.002	0.001	0.06

* Coefficient and SEM represent changes in plasma lipids (mg dL^−1^) corresponding to 1% increase in DNA methylation adjusing for the covariates below.

† *P*:*P*-value for the association between DNA methylation (%) and plasma lipids adjusting for pedigree, sex, center, and the first principal component of cellular purity and population structure.

‡ Coefficient and SEM represent changes in log-transformed TG (mg dL^−1^) corresponding to 1% increase in DNA methylation adjusing for the covariates below.

**Figure 2:**
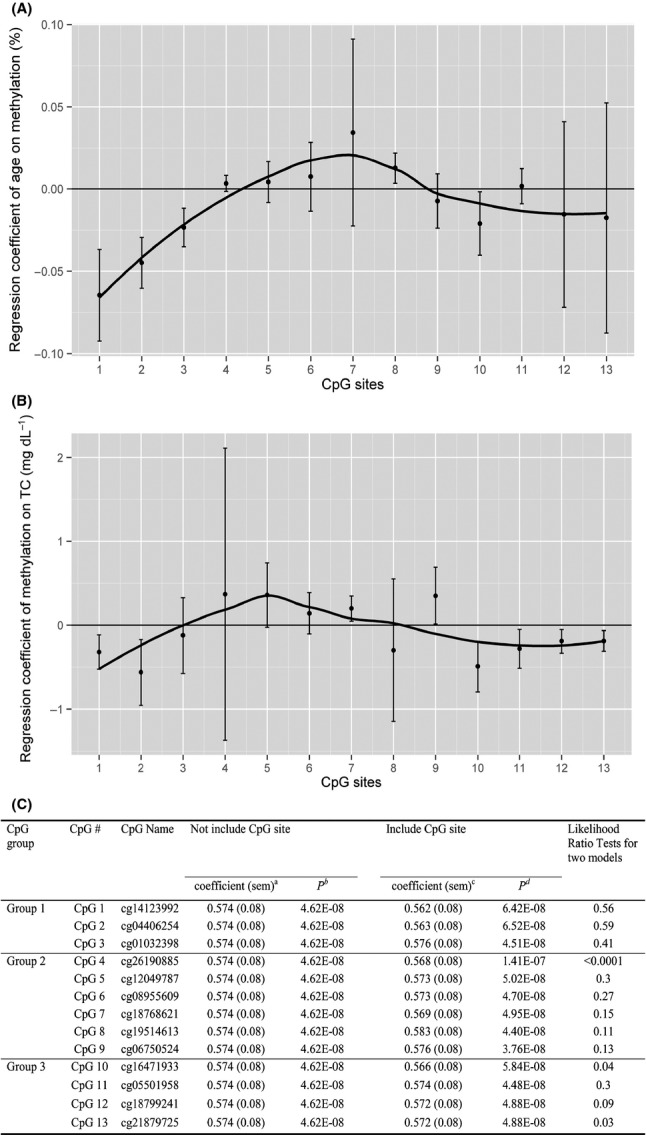
*APOE* methylation patterns may act as the intermediate factors of the effects of age on blood lipids. Panels A and B provided qualitative evidence by listing 13 CpG sites as x-axis and the y-axis are the regression coeffiecients of age for the outcome of methylation (%) (A) and the regression coefficients of methylation for the outcome of TC (mg dL^−1^) (B). Black dot represents point estimate of regression coefficient for each CpG site from generalized linear models adjusting for pedigree, gender, study center, and the first principal component of cellular purity and population structure. Lower and upper bars represent the lower and higher values of 95% confidence intervals for each coefficient. Black line is the fitted line for the pattern of all 13 CpG sites. Panel C provided quantitative evidence based on the likelihood ratio test of the differences in the regression coefficients of the effects of age on TC yielded by the model without and with the adjustment of *APOE* methylation. ^a^Coefficient and SEM, and ^b^*P* represent the magnitude and significance of the changes in blood total cholesterol (mg dL^−1^) corresponding to 1 year increase in age adjusting for pedigree, sex, center, and the first principal component of cellular purity and population structure; ^c^Coefficient and SEM, and ^d^*P* represent the magnitude and significance of the changes in blood total cholesterol (mg dL^−1^) corresponding to 1 year increase in age with further adjustment for the methylation levels of each CpG site at *APOE* locus in addition to pedigree, sex, center, and the first principal component of cellular purity and population structure.

Based on the observed mutual associations among *APOE* methylation patterns, age, and plasma lipids, we proposed that the effect of age on blood lipids may partially contributed by the changes in *APOE* methylation patterns. To test this hypothesis, we first conducted a qualitative analysis by plotting the regression coefficients representing the effects of age on methylation of each CpG site (Fig.2A) and the regression coefficients representing the effects of methylation of each CpG site on TC (Fig.2B). The pattern of the association between age and methylation is moderately parallel to that between methylation and TC, which showed the negative associations for CpG sites in both Group 1 and Group 3, both of which demonstrated hypermethylation, and a positive association for CpG sites in Group 2 which showed hypomethylation. Next, we performed a quantitative analysis to test whether *APOE* methylation patterns are the intermediate factor between age and TC. We found that regression coefficients of the effect of age on TC became smaller after the regression model included the adjustment for methylation levels of almost all the CpG sites except for site 3 (cg01032398). Also, the likelihood ratio test showed that three CpG sites play a statistically significant role as the intermediate factor between age and TC, which were CpG 4 (cg26190885) (*P *<* *0.0001), CpG 10 (cg16471933) (*P *=* *0.04), and CpG 13 (cg21879725) (*P *=* *0.03).

### *APOE* methylation patterns are associated with *APOE* genetic variants

We next explored relationships between *APOE* methylation and *APOE* sequence variants. Two CpG sites in Group 2 and Group 3 were significantly associated with *APOE* ε variants (Table5). For CpG 9 (cg06750524) and CpG 10 (cg16471933), the order for the methylation level is ε2 carriers < ε3/ε3 < ε4 carriers (*P *=* *3.51E-05 and 0.03, respectively). Two other CpG sites had a borderline significance (*P *=* *0.05), which were CpG 8 (cg19514613) and CpG 13 (cg21879725).

**Table 5:** *APOE* methylation patterns by genetic variants in GOLDN*

			*APOE* rs405509				*APOE* ε variants			
			CC	AC	AA		ε2 carriers	ε3/ ε3	ε4 carriers	
CpG group	CpG #	CpG name	*n *= 256	*n *= 500	*n *= 236	*P* †	*n *= 99	*n *= 588	*n *= 257	*P* †
Group 1	CpG 1	cg14123992	85.87 (0.56)	86.19 (0.35)	85.52 (0.62)	0.61	86.84 (0.32)	85.70 (0.41)	86.14 (0.49)	0.84
	CpG 2	cg04406254	69.96 (0.37)	71.07 (0.18)	71.10 (0.26)	0.02	70.36 (0.34)	70.84 (0.20)	70.85 (0.30)	0.42
	CpG 3	cg01032398	81.80 (0.19)	81.98 (0.22)	82.23 (0.21)	0.19	82.07 (0.30)	81.78 (0.19)	82.43 (0.21)	0.07
Group 2	CpG 4	cg26190885	9.22 (0.08)	9.34 (0.05)	9.35 (0.08)	0.23	9.28 (0.11)	9.38 (0.05)	9.23 (0.07)	0.29
	CpG 5	cg12049787	6.97 (0.30)	6.87 (0.18)	6.89 (0.28)	0.85	6.57 (0.12)	7.01 (0.18)	6.83 (0.28)	0.87
	CpG 6	cg08955609	5.11 (0.57)	4.61 (0.31)	4.31 (0.38)	0.26	4.00 (0.08)	4.80 (0.32)	4.69 (0.47)	0.55
	CpG 7	cg18768621	13.45 (0.99)	13.05 (0.60)	11.93 (0.74)	0.24	13.56 (1.87)	13.37 (0.60)	11.51 (0.59)	0.11
	CpG 8	cg19514613	13.20 (0.17)	13.52 (0.11)	13.61 (0.14)	0.06	13.17 (0.29)	13.37 (0.11)	13.72 (0.13)	0.05
	CpG 9	cg06750524	24.41 (0.38)	25.52 (0.21)	25.44 (0.26)	0.03	23.85 (0.29)	25.05 (0.19)	26.25 (0.33)	3.51E-05
Group 3	CpG 10	cg16471933	79.53 (0.37)	80.02 (0.28)	80.78 (0.30)	0.01	79.07 (0.57)	80.07 (0.26)	80.46 (0.29)	0.03
	CpG 11	cg05501958	95.28 (0.49)	95.71 (0.24)	96.15 (0.10)	0.09	96.15 (0.11)	95.67 (0.24)	95.58 (0.38)	0.41
	CpG 12	cg18799241	85.34 (0.99)	85.69 (0.62)	86.84 (0.74)	0.24	85.15 (1.80)	85.45 (0.61)	87.06 (0.58)	0.13
	CpG 13	cg21879725	82.35 (1.12)	82.41 (0.72)	84.45 (0.82)	0.14	81.57 (2.16)	82.21 (0.72)	84.68 (0.66)	0.05

* Data are the least square means (standard error of the means) of *APOE* methylation (%) adjusted for the covariates below.

† *P*:*P*-value for the association between *APOE* methylation (%) and genetic variants adjusting for pedigree, sex, center, and the first principal components of cellular purity and population structure.

Promoter SNP rs405509 had significant associations with methylation of CpG 2 (cg04406254) in Group 1, CpG 9 (cg06750524) in Group 2, and CpG 10 in Group 3 (cg16471933) (*P *=* *0.02, 0.03, and 0.01, respectively) (Table5). Homozygotes of the minor allele (AA) had the highest methylation levels for these three CpG sites, while the homozygotes of major allele (CC) had the lowest, with the values for the heterozygotes in the middle.

### *APOE* methylation patterns are modulated by the interactions between age and *APOE* genetic variants

**Figure 3:**
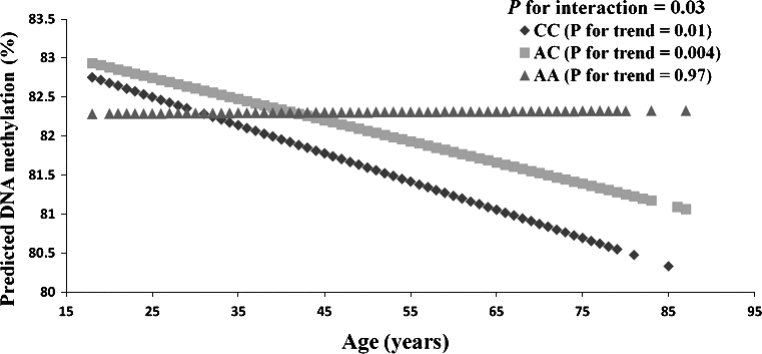
Interaction between rs405509 and age for methylation of CpG 3 (cg01032398) in GOLDN. Predicted methylation levels of cg01032398 were calculated based on the regression coefficient of the exposure variable (age) for the outcome (methylation) with the adjustment for the covariates of pedigree, sex, center, and the first principle component of cellular purity and population structure. Predicted methylation levels of cg01032398 by genotype of rs405509 were plotted against age, adjusted for pedigree, gender, center, and the first principal component of cellular purity and population structure. *P*-values indicate the statistical significance of the adjusted interaction term and adjusted regression coefficients in the regression line corresponding to three genotype groups of rs405509 (diamond for CC, square for AC, and triangle for AA).

Finally, we examined genetic variants as potential modulators of the effect of age on *APOE* methylation. Promoter SNP rs405509 significantly interacted with age to modulate methylation of CpG 3 (cg01032398) (*P* for interaction = 0.03), a CpG site in Group 1 (Fig.3). For major allele carriers (CC and AC), older age was significantly associated with lower methylation of CpG 3 (*P* for trend = 0.01 and 0.004, respectively). However, there was no association between age and methylation of CpG 3 in homozygotes for the minor allele (AA) (*P* for trend = 0.97).

## Discussion

In the current study, we described the methylation patterns of the *APOE* locus and their correlations with gene expression and observed associations between *APOE* methylation and age, blood lipids, and sequence variants, as well as an interaction between age and a methylation-associated promoter variant. This is the first study to explore *APOE* methylation at single nucleotide resolution in a population of almost a thousand individuals.

Our finding that age is associated with *APOE* methylation is consistent with a previous study by Wang et.al. (Wang *et al*., 2008), showing that the differences in *APOE* methylation between individuals with and without Alzheimer's disease increases with age. Other studies demonstrated that age affects global DNA methylation (Fraga *et al*., 2005). Compared to younger monozygous twins, older twins exhibited greater differences in DNA methylation. This may be a consequence of the fact that DNA methylation is modifiable by environmental factors (Cedar & Bergman, 2012), which accumulate gradually or change continuously with age. In our study, we expected *APOE* methylation to change with age because *APOE* is considered an age-related gene. This is based on the genetic associations between *APOE* variants and many age-related diseases, such as coronary heart disease (Ward *et al*., 2009), atherosclerosis (Davignon *et al*., 1988), age-related macular degeneration (AMD) (Klaver *et al*., 1998), and Alzheimer's disease (Saunders *et al*., 1996). We also observed that the direction of the age-associated differences in methylation levels appeared to be associated with the existing degree of methylation. Specifically, we observed that greater age was associated with less methylation in hypermethylated regions but with greater methylation in hypomethylated regions. Finally, we obtained both qualitative and quantitative evidence to support the potential biological connections between age, *APOE* methylation, and plasma lipids by indicating that the effects of age on plasma lipids may partially act through *APOE* methylation. However, the magnitude of the effect of *APOE* methylation is small, which may be related to the tiny contribution of the single *APOE* gene to the entire genome-wide effects of the age on DNA methylation.

Two hypothetical mechanisms for the observed differences in *APOE* methylation by *APOE* variants are differential allelic gene expression and changes in the DNA sequence that affect CpG site formation. With respect to the first mechanism, the minor A allele of the promoter SNP rs405509 has been reported to exhibit lower gene transcription compared to the major C allele (Artiga *et al*., 1998). For the *APOE* ε variants, mRNA expression for ε3 allele was shown to be greater than that of ε4 (Lambert *et al*., 1997). Based on the established relationship between DNA methylation and reduced gene expression (Lindahl, 1981), our findings of higher methylation levels for carriers of the rs405509 A allele and ε4 allele in GOLDN are consistent with the lower expression reported in previous studies. The second potential mechanism by which genotype may alter methylation is based on the creation and disruption of CpG sites by nucleotide changes that determine the *APOE* ε variants. The two SNPs that constitute *APOE* ε variants are both CpG-related SNPs. That means that individuals with ε3/ε3 genotype have 2 CpG sites created by the homozygous C alleles for rs7412, ε2 carriers have either 0 or 1 CpG site created by the heterozygous C allele for rs7412, and ε4 carriers have either 3 or 4 CpG sites created by the homozygous C alleles of rs7412 and the heterozygous or the homozygous C alleles of rs429358. Based on the recognition that most DNA methylation in the mammalian genome occurs on CpG sites (Lister *et al*., 2009), the density of CpG sites is likely to affect the local DNA methylation patterns. The fact that ε4 carriers have the greatest number of CpG sites while ε2 carriers have the smallest number with ε3/ε3 in the middle is consistent with our finding that methylation levels for most CpG sites are in the order of ε4 carriers > ε3/ε3 > ε2 carriers.

In light of our findings that both age and *APOE* genotype were related to methylation, a logical next step was to examine whether these two factors might act in combination to alter methylation. Our observation that promoter SNP rs405509 significantly modified the association between age and promoter methylation is novel and plausible. Greater age was associated with less promoter methylation in the carriers of the major allele (CC and AC) but not in the homozygotes of the minor allele (AA), such that methylation of those in the AA group remained high regardless of age. This age-related allelic difference in methylation level may provide mechanistic support for previous findings linking the A allele of rs405509 to greater risk of myocardial infarction (Lambert *et al*., 2000), premature coronary heart disease (Viitanen *et al*., 2001), and Alzheimer's disease (Lambert *et al*., 1998), but lower plasma concentration of ApoE (Lambert *et al*., 2000).

This study had a number of limitations. Based on the cross-sectional study design, we cannot establish causality. Further mechanistic studies are necessary. The measurement of *APOE* methylation in circulating CD4^+^ T lymphocytes may provide a limited perspective on a protein with multiple functions. However, the observed consistent methylation patterns across different cell types increases the generalizability of our findings. Also, measurements of *APOE* in circulating cells may not provide direct evidence for age-related diseases, but circulating *APOE* could provide indirect evidence through their demonstrated effects on plasma lipids. In addition, circulating *APOE* may act as a biomarker of the changes in the neuronal systems based on our findings of the highly similar methylation patterns between blood T lymphocytes and brain tissues. Finally, the selected 13 CpG sites may not represent the methylation pattern of the whole *APOE*, but the usual highly correlated methylation status of the nearby CpG sites (Shoemaker *et al*., 2010) and the consistent methylation patterns between our observation and previous publication with higher coverage may ameliorate this limitation to some extent.

In summary, we characterized thirteen CpG sites at the *APOE* locus into three groups based on their genetic locations and methylation status measured in both primary T cells in GOLDN and a wide array of other cell types obtained through ENCODE and previous publications and observed that most of these sites were negatively correlated with *APOE* gene expression based on ENCODE data. With a large population in GOLDN, we found that age was indeed associated with *APOE* methylation and linked those associations to changes in plasma lipid profile. Furthermore, we observed that methylation-associated genetic variants of *APOE* modified the aging effect on methylation. Our findings are novel and consistent with the previous evidence from genetic studies and may provide potential mechanistic explanations for aging-related functions of *APOE*.

## Experimental procedures

### Study population

The GOLDN study was designed to evaluate genetic factors that modulate plasma lipid responses to a diet intervention (consumption of a high-fat meal) and fenofibrate treatment. GOLDN participants are of European ancestry and were enrolled from the National Heart, Lung, and Blood Institute Family Heart Study (Higgins *et al*., 1996). This GOLDN analysis included 475 men and 518 women (age ranged from 18 to 87 years) who have baseline data for all required variables. The detailed design and methodology of GOLDN has been described (Corella *et al*., 2007). Of relevance for this analysis, GOLDN required all subjects with a history of antilipemic drug use to be off all antilipemic medications for at least 4 weeks prior to their study visit. The protocol for this study was approved by the Human Studies Committee of Institutional Review Board at the University of Minnesota, University of Utah, and Tufts University/New England Medical Center. Written informed consent was obtained from all participants. Fasting blood samples were collected to measure the lipid profile, and detailed methodology was described previously (Tsai *et al*., 2005). Dietary intake was estimated using the diet history questionnaire (Subar *et al*., 2001; Thompson *et al*., 2002). Physical activity was assessed by a questionnaire containing questions on the number of hours per day dedicated to different levels (heavy, slight, and sedentary) of activity (Corella *et al*., 2011).

### Genotyping and methylation measurements

Genomic DNA for sequence genotyping was extracted from blood samples using Gentra Puregene Blood Kits (Gentra Systems, Inc., Minneapolis, MN, USA). Genotypes of 3 SNPs included in the current study, which were rs405509, rs429358, and rs7412, were obtained using TaqMan assays on a ABI 7900HT system (Applied Biosystems, Foster City, CA, USA). *APOE* genotypes were called on the basis of the guidelines of Hixson and Vernier (Hixson & Vernier, 1990) according to the genotypes of rs429358 and rs7412.

Detailed methodology to measure DNA methylation was described previously (Absher *et al*., 2013). CD4^+^ T cells for methylation measurement were extracted from baseline frozen buffy coat samples isolated from peripheral blood using positive selection (Invitrogen, Grand Island, NY, USA) followed by sorting of subsets by flow cytometry (FACSAriaII, BD Biosciences, San Jose, CA, USA). Cells were then lysed, and DNA was extracted using QIAGEN DNAeasy kits (QIAGEN, Germantown, MD, USA). DNA sample (500 ng) was treated with sodium bisulfite using Zymo EZ DNA methylation kit (Zymo Research Corporation, Irvine, CA, USA). DNA methylation was measured by the Infinium Human Methylation 450K BeadChip (Illumina, San Diego, CA, USA) through amplification, hybridization, and imaging steps. Intensity files were generated and analyzed with Illumina's GenomeStudio, through which beta scores and ‘detection *P*-values’ were generated. These beta scores represent the proportion of total signal from the methylation-specific probe or color channel. The ‘detection *P*-values’ were defined as the probability that the total intensity for a given probe falls within the background signal intensity. Those CpG probes with ‘detection *P*-values’ greater than 0.01 and with more than 10% of samples that failed to yield adequate intensity were eliminated from further analysis. Those samples with more than 1.5% missing data points across ∼470,000 autosomal CpGs were removed. After quality control, 13 CpG sites related to *APOE* remained. Starting from the 5′, the first four CpG sites (cg14123992, cg04406254, cg01032398, and cg26190885) were located within 1.5 kb before the transcription start site, the fifth CpG site (cg12049787) was within the first exon, the next three CpG sites (cg08955609, cg18768621, and cg19514613) were within the first intron, the ninth CpG site (cg06750524) was within the second intron, and the last four CpG sites (cg16471933, cg05501958, cg18799241, and cg21879725) were within the fourth exon.

### DNA methylation datasets

Methylation levels of all 13 CpG sites for 61 cell lines and one primary liver cell in ENCODE were downloaded (September 20, 2013) from UCSC genome browser HAIB Methyl450 track (http://hgdownload.cse.ucsc.edu/goldenPath/hg19/encodeDCC/wgEncodeHaibMethyl450/) and represented by a heat map using R packages of ‘gplots’ (http://cran.r-project.org/web/packages/gplots/index.html) and ‘RColorBrewer’ (http://cran.r-project.org/web/packages/RColorBrewer/index.html). The score of the methylation value associated with each CpG site was defined as the beta value multiplied by 1000, with the beta value in turn defined as the proportion of the intensity value from the methylated bead type from the sum of the intensity values from both methylated and unmethylated bead type plus 100.

Published methylation data of primary cells from tissues of brain (Day *et al*., 2013; Kozlenkov *et al*., 2014; Wockner *et al*., 2014), muscle (Ribel-Madsen *et al*., 2012; Day *et al*., 2013; Zykovich *et al*., 2013), fat (Ribel-Madsen *et al*., 2012; Grundberg *et al*., 2013), and saliva (Liu *et al*., 2010; Bocklandt *et al*., 2011; Souren *et al*., 2013; Park *et al*., 2014) were obtained through Gene Expression Omnibus (GEO) (http://www.ncbi.nlm.nih.gov/geo/) (Edgar *et al*., 2002) and ArrayExpress (http://www.ebi.ac.uk/arrayexpress/) (Rustici *et al*., 2012).

### Gene expression in ENCODE

Gene expression data for *APOE* in 17 cell lines, which also had methylation data available, in ENCODE were downloaded (11-08-2013) from UCSC genome browser Duke Affymetrix Exon Array track (http://hgdownload.cse.ucsc.edu/goldenPath/hg19/encodeDCC/wgEncodeDukeAffyExon/). This track displayed exon array data that had been aggregated to the gene level for those probes that had been linked to genes. The expression score for each cell line represented a linearly scaled value for that particular cell type multiplied by 100 and ranged from 0 to 1000.

### Statistical methods

In ENCODE data, Pearson's correlation analysis was conducted to test the correlations between methylation of each CpG site and gene expression of *APOE*. In GOLDN, Mantel–Haenszel χ^2^ tests and ANOVA tests were used to examine the trend of significance in characteristics of the study population, as categorical and continuous variables, respectively, by age, categorized in quintiles. Generalized linear models were used to test the association of methylation of each CpG site with age (continuous variable), blood lipids, and *APOE* genotypes, and the interaction between age (continuous variable) and selected variants. Each CpG site was included into the model separately. *APOE* ε variants were coded into three categories, ε3/ε3, ε2 carriers (including ε2/ε2 and ε2/ε3), and ε4 carriers (including ε4/ε4 and ε3/ε4). Individuals with the ε2/ ε4 genotype (*n *= 46) were excluded from the analysis to distinguish the distinct role of each variant. The primary analysis was adjusted for pedigree, sex, study center, and the first principal component of both cellular purity (Frazier-Wood *et al*., 2014) and population structure. To test the effect of other potential confounders, the secondary analysis was adjusted for smoking (never smoker, past smoker, and current smoker), drinking (ever drink alcohol or not), total energy intake (kcal day^−1^), physical activity (hours of total physical activity day^−1^), vitamin B12 intake (μg day^−1^), folate intake (μg day^−1^), hormone replacement therapy in women, a history of taking antilipemic medication, and time of fasting blood drawn (am or pm) (Reddy & Reddy, 1990; Armstrong *et al*., 2013). Likelihood ratio tests were conducted to analyze whether the effects of age on plasma TC is partially through methylation of *APOE*. Each continuous variable was tested for normality, and log-transformations were performed for those variables not following a normal distribution. Correlation analysis with data from ENCODE consortium and all data analysis with GOLDN population were performed using sas (version 9.3 for Windows; SAS Institute, Inc. Cary, NC, USA). Considering the main hypothesis is the general pattern of *APOE* methylation rather than the specific CpG site, a two-tailed *P*-value of < 0.05 was considered statistically significant.

## Supporting Information

**Table S1** Cell types and tissues included in the current study.

**Fig. S1** Heat map of *APOE* methylation patterns measured in the blood T lymphocyte in GOLDN. The pink represents hypermethylation (> 50%), the green represents hypomethylation (< 50%), and the gray represents missing value.

**Fig. S2** Heat map of *APOE* methylation patterns measured in the primary brain tissue. The pink represents hypermethylation (> 50%), the green represents hypomethylation (< 50%), and the gray represents missing value.

**Fig. S3** Heat map of *APOE* methylation patterns measured in the primary saliva tissue. The pink represents hypermethylation (> 50%), the green represents hypomethylation (< 50%), and the gray represents missing value.

**Fig. S4** Heat map of *APOE* methylation patterns measured in the primary adipose tissue. The pink represents hypermethylation (> 50%), the green represents hypomethylation (< 50%), and the gray represents missing value.

**Fig. S5** Heat map of *APOE* methylation patterns measured in the primary muscle. The pink represents hypermethylation (> 50%), the green represents hypomethylation (< 50%), and the gray represents missing value.

**Fig. S6** Heat map of *APOE* methylation patterns measured in primary hepatocyte and 61 cell lines in ENCODE. The pink represents hypermethylation (> 50%), the green represents hypomethylation (< 50%), and the gray represents missing value.
