# Supplementary material for: Genetic variants modify the effect of age on APOE methylation in the Genetics of Lipid Lowering Drugs and Diet Network study
Source: Aging Cell. 2014 Dec 4;14(1):49–59. doi: 10.1111/acel.12293 (PMC4324456; doi:10.1111/acel.12293)
Supplement: Supplementary file 1 [file acel0014-0049-sd1.docx]

| Supplementary Table 1. Cell types and tissues included in the current study | | | | |
| --- | --- | --- | --- | --- |
| Tissue | Cell type | Cell name | Cell description | Reference / Resource |
| Brain | primary cells | / | / | Wockner, 2014; Kozlenkov, 2014; Day, 2013 |
|  | cell lines | BE2_C | Neuroblastoma | ENCODE |
|  |  | NH-A | Astrocytes | ENCODE |
|  |  | PFSK-1 | Neuroectodermal cell line | ENCODE |
|  |  | SK-N-MC | Neuroepithelioma cell line | ENCODE |
|  |  | SK-N-SH | Neuroblastoma | ENCODE |
|  |  | SK-N-SH_RA | Neuroepithelioma cell line differentiated with retinoic acid | ENCODE |
|  |  | U87 | Glioblastoma | ENCODE |
| Saliva | primary cells | / | / | Liu, 2010; Bocklandt, 2011; Souren, 2013; Park, 2014 |
| Fat | primary cells | / | subcutaneous adipose tissue | Ribel-Madsen, 2012; Grundberg, 2013 |
| Muscle | primary cells | / | skeletal muscle | Ribel-Madsen, 2012; Day, 2013; Zykovich, 2014 |
| Blood | cell lines | CMK | Acute megakaryocytic leukemia cells | ENCODE |
|  | cell lines | GM06990 | B lymphocyte | ENCODE |
|  | cell lines | GM12878 | B lymphocyte | ENCODE |
|  | cell lines | GM12891 | B lymphocyte | ENCODE |
|  | cell lines | GM12892 | B lymphocyte | ENCODE |
|  | cell lines | GM19239 | B lymphocyte | ENCODE |
|  | cell lines | HL60 | Promyelocytic leukemia cells | ENCODE |
|  | cell lines | Jurkat | T lymphoblastoid | ENCODE |
|  | cell lines | K562 | Leukemia | ENCODE |
|  | cell lines | NB4 | Acute promyelocytic leukemia cell line | ENCODE |
| Breast | cell lines | MCF-7 | Mammary gland adenocarcinoma | ENCODE |
|  | cell lines | Mcf10a | Mammary gland non-tumorigenic epithelial inducible cell line | ENCODE |
|  | cell lines | Mcf10a_Tam | Mammary gland non-tumorigenic epithelial inducible cell line treated with tamoxifen | ENCODE |
|  | cell lines | T-47D_DMSO | Mammary ductal carcinoma epithelial cell line treated with DMSO | ENCODE |
|  | cell lines | HMEC | Mammary epithelial cells | ENCODE |
|  | cell lines | HMEC | Mammary epithelial cells | ENCODE |
| Colon | cell lines | Caco-2 | Colorectal adenocarcinoma | ENCODE |
|  | cell lines | HCT-116_HAIB | Colorectal carcinoma from HudsonAlpha Institute | ENCODE |
|  | cell lines | HCT-116_Stanford | Colorectal carcinoma from Standford University | ENCODE |
| Epithelia | cell lines | HAEpiC | Amniotic epithelial cells | ENCODE |
|  | cell lines | HCPEpiC | Choroid plexus epithelial cells | ENCODE |
|  | cell lines | HEEpiC | Esophageal epithelial cells | ENCODE |
|  | cell lines | HIPEpic | Iris pigment epithelial cells | ENCODE |
|  | cell lines | HNPCEpiC | Non-pigment ciliary epithelial cells | ENCODE |
|  | cell lines | HPAEpiC | Pulmonary alveolar epithelial cells | ENCODE |
|  | cell lines | HRCEpiC | Renal cortical epithelial cells | ENCODE |
|  | cell lines | HRE | Renal epithelial cells | ENCODE |
|  | cell lines | HRPEpiC | Retinal pigment epithelial cells | ENCODE |
|  | cell lines | NHBE | Bronchial epithelial cells | ENCODE |
|  | cell lines | RPTEC | Renal proximal tubule epithelial cells | ENCODE |
|  | cell lines | SAEC | Small airway epithelial cells | ENCODE |
| Skin | cell lines | AG04449 | Fetal buttock/thigh fibroblast | ENCODE |
|  | cell lines | AG09309 | Adult toe fibroblast | ENCODE |
|  | cell lines | AG10803 | Abdominal skin fibroblasts | ENCODE |
|  | cell lines | BJ | Skin fibroblast | ENCODE |
|  | cell lines | NHDF-neo | Neonatal dermal fibroblasts | ENCODE |
|  | cell lines | ProgFib | Fibroblasts | ENCODE |
| Others | cell lines | AoSMC | Aortic smooth muscle cells | ENCODE |
|  | cell lines | HUVEC | Umbilical vein endothelial cells | ENCODE |
|  | cell lines | Hela-S3 | Cervical carcinoma | ENCODE |
|  | cell lines | H1-hESC | Embryonic stem cells | ENCODE |
|  | cell lines | AG09319 | Gum tissue fibrolasts | ENCODE |
|  | cell lines | HCF | Cardiac fibrolasts | ENCODE |
|  | cell lines | HCM | Cardiac myocytes | ENCODE |
|  | cell lines | HEK293 | Embryonic kidney cells | ENCODE |
|  | primary cells | Hepatocyte | Primary hepatocyte | ENCODE |
|  | cell lines | HepG2 | Hepatocellular carcinoma | ENCODE |
|  | cell lines | ECC-1 | Epithelial cell line | ENCODE |
|  | cell lines | AG04450 | Fetal lung fibroblast | ENCODE |
|  | cell lines | IMR90 | Fetal lung fibroblasts | ENCODE |
|  | cell lines | SKMC | Skeletal muscle cells | ENCODE |
|  | cell lines | Ovcar-3 | Ovarian adenocarcinoma | ENCODE |
|  | cell lines | PANC-1 | Pancreatic carcinoma | ENCODE |
|  | cell lines | LNCaP | Prostate adenocarcinoma | ENCODE |
|  | cell lines | PrEC | Prostate epithelial cell line | ENCODE |
|  | cell lines | NT2-D1 | Malignant pluripotent embryonal carcinoma | ENCODE |
