# Supplementary figures and images for: Genetic variants modify the effect of age on APOE methylation in the Genetics of Lipid Lowering Drugs and Diet Network study
Source: Aging Cell. 2014 Dec 4;14(1):49–59. doi: 10.1111/acel.12293 (PMC4324456; doi:10.1111/acel.12293)

Color Key

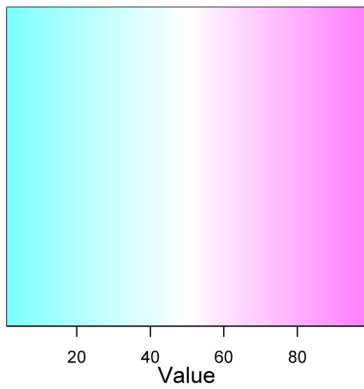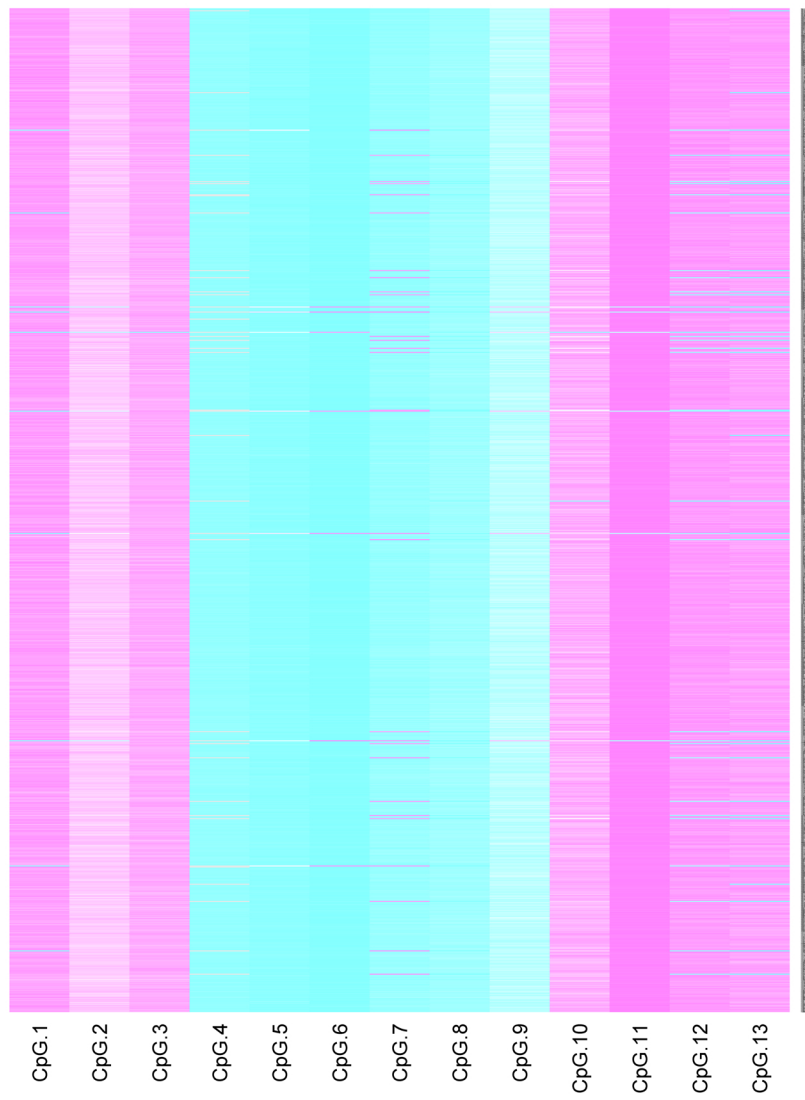

Supplement: Supplementary file 2 [file acel0014-0049-sd2.pdf]

Color Key

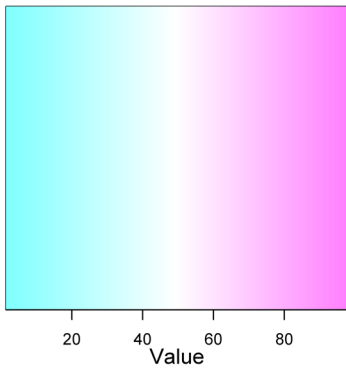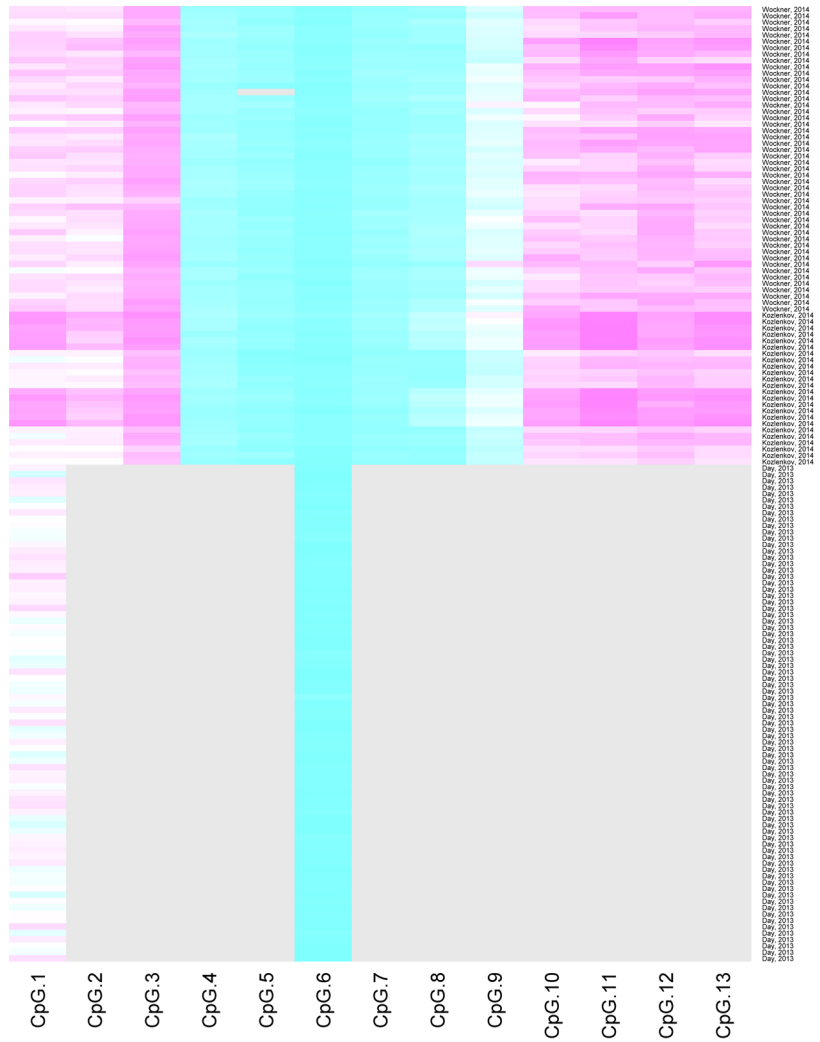

Supplement: Supplementary file 3 [file acel0014-0049-sd3.pdf]

Color Key

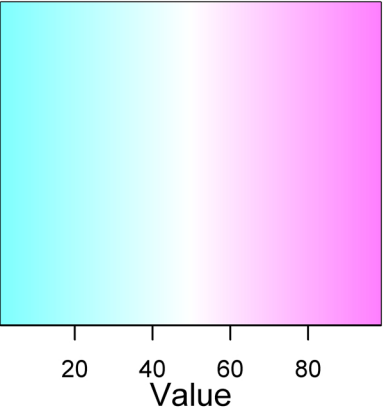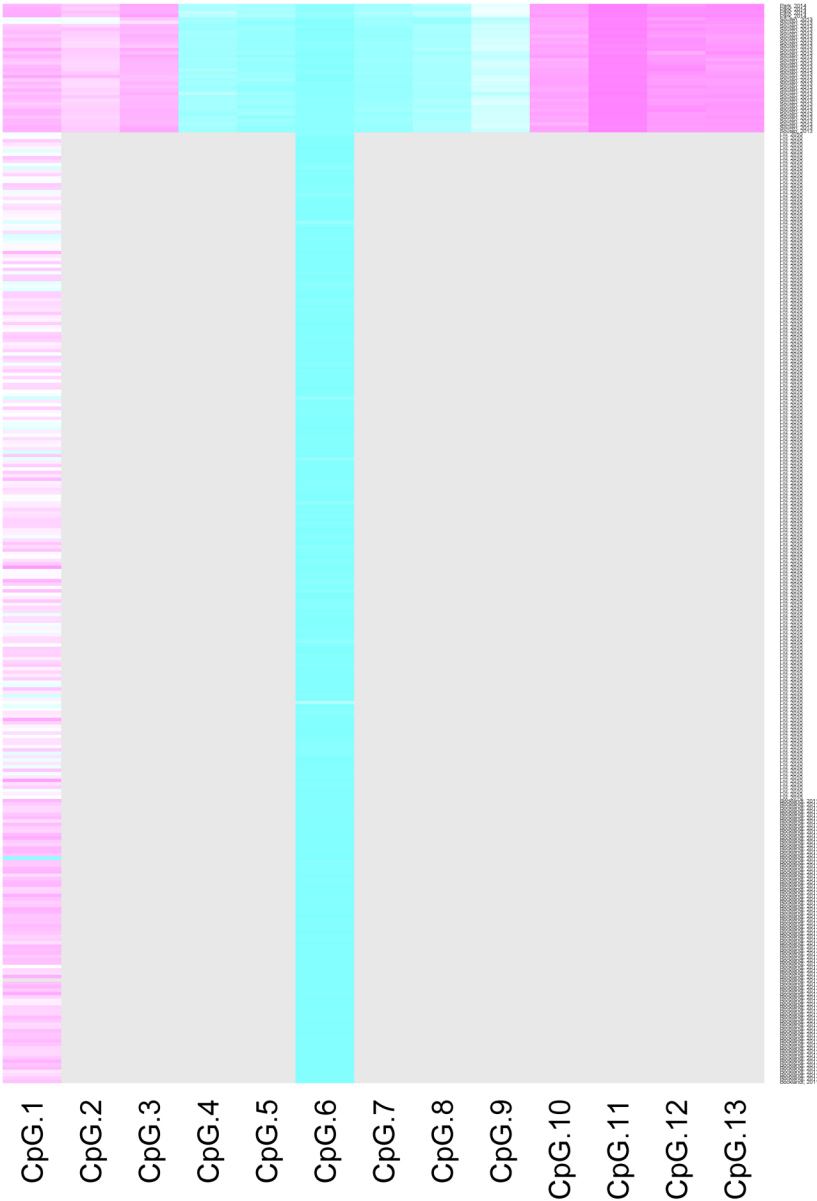

Supplement: Supplementary file 4 [file acel0014-0049-sd4.pdf]

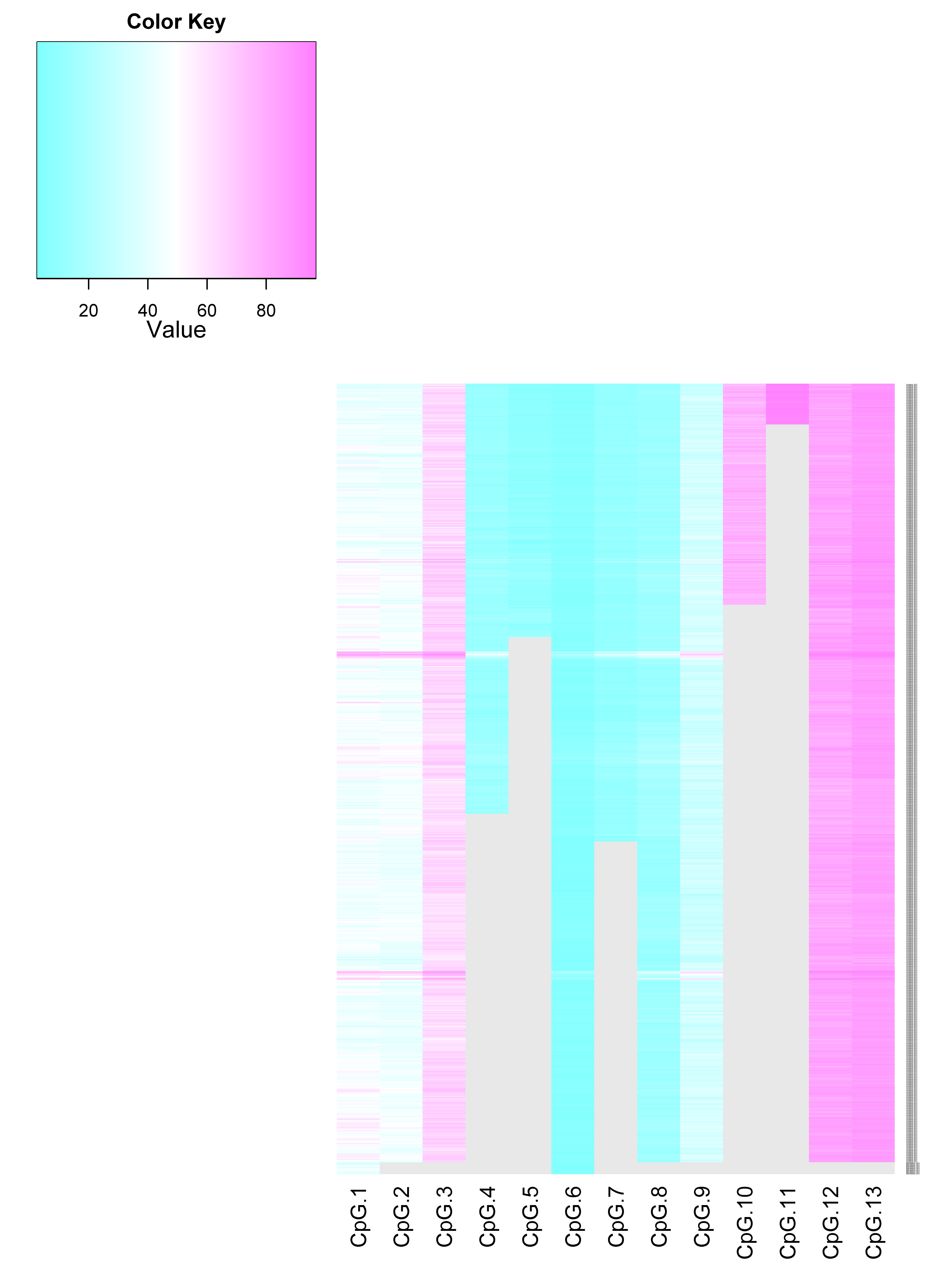

Supplement: Supplementary file 5 [file acel0014-0049-sd5.jpg]

Color Key

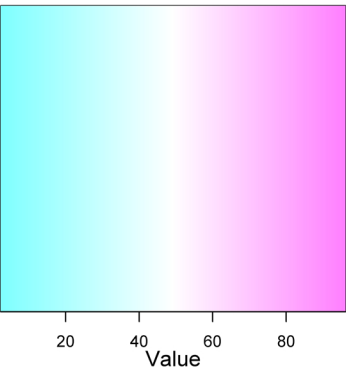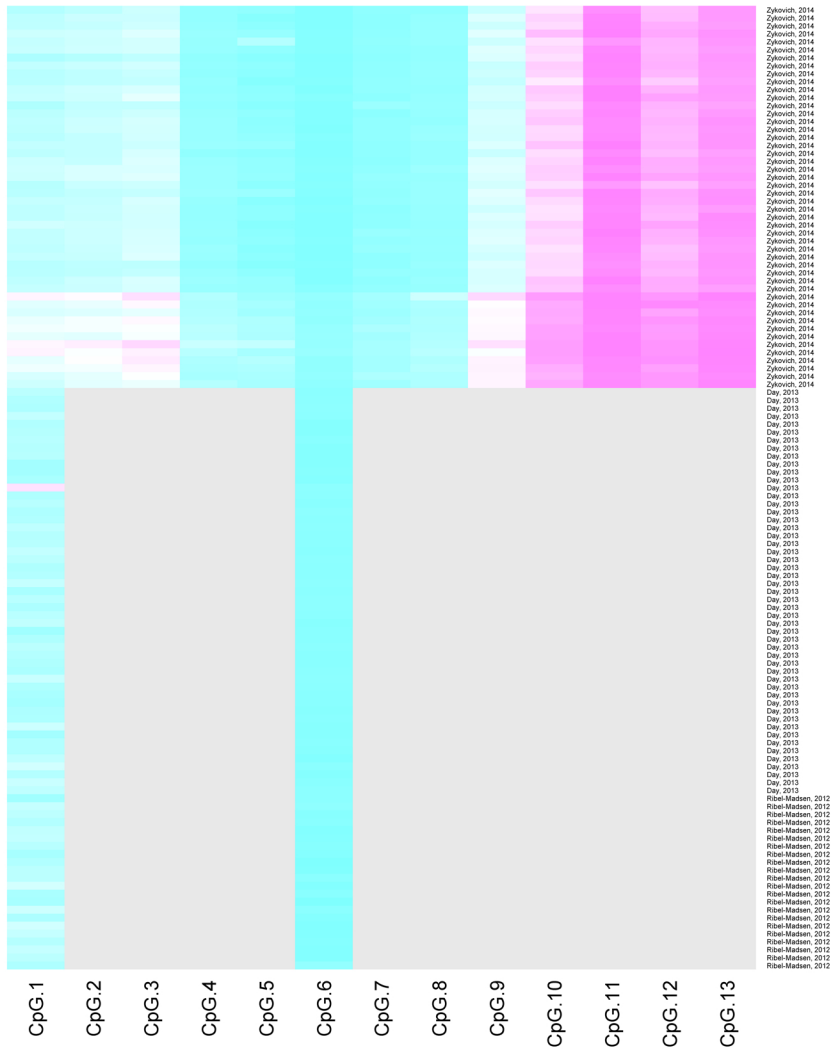

Supplement: Supplementary file 6 [file acel0014-0049-sd6.pdf]

Color Key

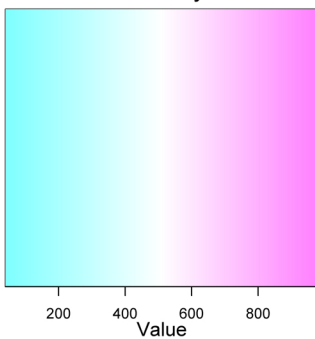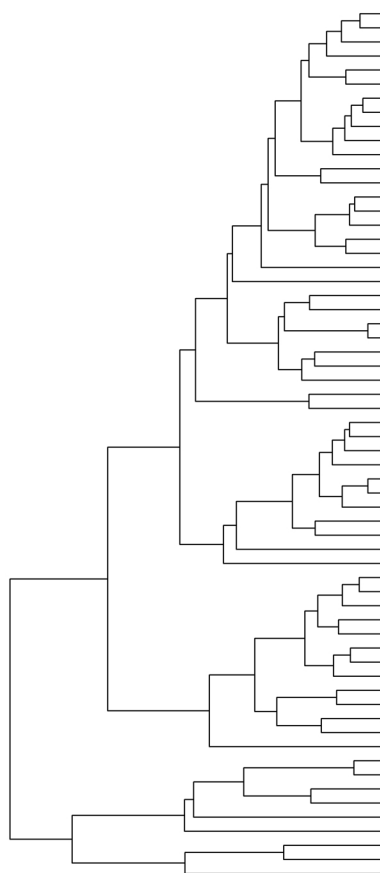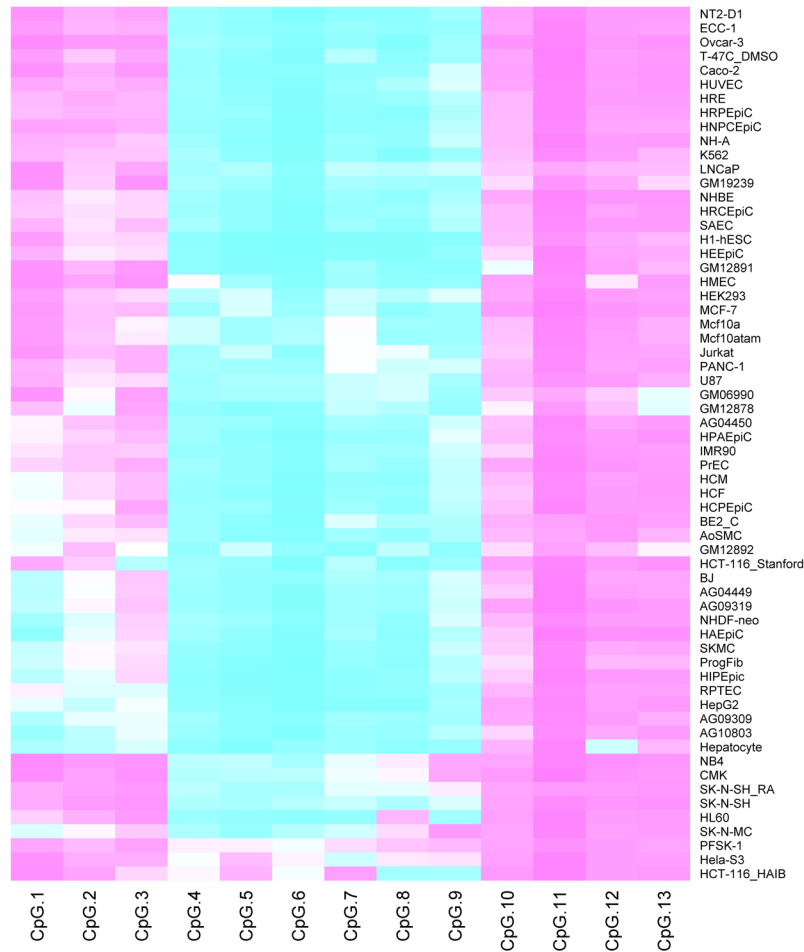

Supplement: Supplementary file 7 [file acel0014-0049-sd7.pdf]
